# *Serratia* spp. as plant growth-promoting bacteria alleviating salinity, drought, and nutrient imbalance stresses

**DOI:** 10.3389/fmicb.2024.1342331

**Published:** 2024-03-18

**Authors:** Iryna Kulkova, Barbara Wróbel, Jakub Dobrzyński

**Affiliations:** Institute of Technology and Life Science – National Research Institute, Raszyn, Poland

**Keywords:** *Serratia* spp., plant growth-promotion bacteria, salinity stress, drought stress, nutritional imbalance stress

## Abstract

In agricultural environments, plants are often exposed to abiotic stresses including temperature extremes, salt stress, drought, and heavy metal soil contamination, which leads to significant economic losses worldwide. Especially salt stress and drought pose serious challenges since they induce ionic toxicity, osmotic stress, and oxidative stress in plants. A potential solution can be the application of bacteria of the *Serratia* spp. known to promote plant growth under normal conditions Thus the mini-review aims to summarize the current knowledge on plant growth promotion by *Serratia* spp. (under the conditions of salinity stress, drought, and nutrient deficit) and highlight areas for development in the field. So far, it has been proven that *Serratia* spp. strains exhibit a variety of traits contributing to enhanced plant growth and stress tolerance, such as phytohormone production, ACC deaminase activity, nitrogen fixation, P and Zn solubilization, antioxidant properties improvement, and modulation of gene expression. Nevertheless, further research on *Serratia* spp. is needed, especially on two subjects: elucidating its mechanisms of action on plants at the molecular level and the effects of *Serratia* spp. on the indigenous soil and plant microbiota and, particularly, the rhizosphere. In both cases, it is advisable to use omics techniques to gain in-depth insights into the issues. Additionally, some strains of *Serratia* spp. may be phytopathogens, therefore studies to rule out this possibility are recommended prior to field trials. It is believed that by improving said knowledge the potential of *Serratia* spp. to stimulate plant growth will increase and strains from the genus will serve as an eco-friendly biofertilizer in sustainable agriculture more often.

## 1 Introduction

In the agricultural environment, plants are often exposed to abiotic stresses such as temperature extremes, salt stress, drought, nutrient deficiencies, and heavy metals contamination of soils. These stressors have a significant impact on global agriculture, causing significant economic losses (Faisol et al., 2022; Hewedy et al., 2022; Suleimenova et al., 2023; Wróbel et al., 2023). Salt stress and drought are among the most damaging environmental stresses, which causes plants to simultaneously develop ionic toxicity, osmotic stress, and oxidative stress (Ma et al., 2020; Kulkova et al., 2023). Therefore, eco-friendly solutions such as plant growth-promoting bacteria are needed (Dobrzyński et al., 2022a, 2023a,b).

*Serratia* spp. is a genus of gram-negative, facultatively anaerobic bacteria belonging to the Enterobacteriaceae family (Grimont and Grimont, 2015). This highly diverse genus grows in a variety of habitats including water, soil, plants, and humans. While some *Serratia* species, in particular *Serratia marcescens* are known to cause opportunistic infections in humans and plant diseases, other strains have gained attention due to their potential as plant growth-promoting bacteria (PGPB) (Hasan et al., 2020; Soenens and Imperial, 2020). *Serratia* spp. strains, as endophytic and rhizospheric microorganisms, when interacting with plants not only contribute positively to stimulating plant growth, increase yields, and improve soil quality under normal conditions, but also can be used in environments exposed to various abiotic and biotic stresses (Verma et al., 2019). Furthermore, plant growth promotion through *Serratia* spp. can reduce dependence on synthetic fertilizers, contributing to sustainable and environmentally friendly agricultural practices (Sood et al., 2019).

Salt- and drought-tolerant bacteria have different mechanisms of tolerance to the abiotic stresses (Gamalero and Glick, 2022). Some of the main mechanisms of *Serratia* spp. promoting plant growth under normal, saline, and drought conditions include: (i) production of phytohormones; (ii) ACC deaminase production; (iii) facilitation of nutrient availability (P or Zn solubilization); (iv) uptake of reactive oxygen species (ROS); (v) osmolytes production; exopolysaccharides (EPS) production; (vi) ion homeostasis in plants; (vii) expression of genes encoding salt and drought stress tolerance (Saikia et al., 2018; Mahdi et al., 2021; Nordstedt and Jones, 2021; Ahmad et al., 2022). In addition to *Serratia* spp., PGPB include, for instance, bacteria of the following genera: *Azotobacter*, *Azospirillum*, *Bacillus*, *Paenibacillus*, *Pseudomonas*, and *Rhizobium* (Gopalakrishnan et al., 2015; Aasfar et al., 2021; Dobrzyński et al., 2021, 2022a,b; Cruz-Hernández et al., 2022; Górska et al., 2024). However, *Serratia* spp., compared to most known bacterial plant growth promoters, is not studied as extensively, particularly with regard to its potential to promote plant growth by modulating the native soil or plant microbiota and, importantly, in the mitigation of plant abiotic stresses including salinity stress, drought stress, and nutritional imbalance stress. Nevertheless, despite the insufficient understanding of the mechanisms governing the modes of action of *Serratia* spp. under stress conditions, some *Serratia* spp. strains have been shown to improve plant growth under salt and drought stress conditions (Table 1).

**Table 1:** Overview of salt and plant drought tolerance mechanisms mediated by *Serratia* spp. strains.

**Strains of *Serratia* spp.**	**Plant species**	**Experiments**	**Salinity/ drought stress**	**PGPB traits**	**Results**	**References**
*S. fonticola* S1T1	Cucumber (*Cucumis sativus* L.)	Greenhouse; pot experiment; isolation and molecular characterization using 16S rRNA; evaluation of plant growth dynamics; determination of leaf relative water content (LRWC), electrolytic leakage from leaves, antioxidant enzymes activity; analyses endogenous abscisic acid (ABA), gene expression using qRT-PCR	200 mM NaCl	Production of phytohormones, solubilization of phosphate, production of siderophores and ACC deaminase	Increase of biomass, root length, shoot length, chlorophyll content and LRWC; decrease of levels of MDA, electrolytic leakage, H_2_0_2_, superoxide anion (SOA) and ABA content; increase of CAT, SOD, gene expression *HKT1, NHX, SOS1*	Moon et al. (2023)
*S. marcescens*	Eggplant (*Solanum melongena*)	Greenhouse; evaluation of plant growth dynamics; SPAD values and lipid peroxidation; mineral element analysis; antioxidant enzymes activity	200 mM NaCl	Production of ACC deaminase	Increase of shoot fresh weight, shoot dry weight, stem length, leaf area and chlorophyll; decrease of the levels of MDA and Na^+^ and Cl^−^; increase of SOD, APX, GR	Turhan et al. (2020)
*S. proteamaculans* ATCC35475	Lettuce (*Lactuca sativa L*)	Greenhouse; evaluation of plant growth dynamics; analysis of inorganic elements and chlorophyll II content; antioxidant enzymes activity	Soil salinity (EC = 1.5 and 7.0 dS m^−1^)	Production of EPS	Increase of fresh weight, dry weight, leaf length, leaf area, chlorophyll content, GR and APX	Han and Lee (2005)
*S. marcescens*	Pepper (*Capsicum annuum*)	*In vitro*; pot experiment; isolation and molecular characterization using 16S rRNA; screening of bacteria for plant growth promoting traits; evaluation of plant growth dynamics; determination of chlorophyll content and proline	60, 120, and 240 mM NaCl; drought stress	Phosphate solubilization, production of siderophore, ammonia, ACC deaminase, EPS	Increase of fresh plant weight, shoot length, primary root length, no. of secondary roots, no. of leaves, dry plant weight; increase of chlorophyll a, chlorophyll b, proline	Maxton et al. (2018)
*S. plymuthica* RR-2-5-10; *S. rhizophila* e-p10	Cucumber (*Cucumis sativum* L.)	*In vitro*; greenhouse; plant growth-promoting traits	Soil salinity (EC =659 mS m^−1^)	HCN, production of IAA, ACC deaminase, protease	Increase of dry weight, plant height and fruit yield.	Egamberdieva et al. (2011)
*S. plymuthica DT8*	Jujube (Ziziphus jujuba)	Pot experiment; isolation and molecular characterization using 16S rRNA; evaluation of drought tolerance and plant growth-promoting traits; evaluation of plant growth dynamics; antioxidant enzyme activity and MDA contents	Drought stress	Phosphate solubilization, nitrogen fixation, production of IAA, ACC deaminase, EPS	Increase of plant height, shoot dry matter weight, root dry matter weight; Decrease of the levels of MDA and ethylene content; Increase of SOD, POD, IAA, ABA, RWC, RAS/RS ratio and soil aggregate stability;	Zhang et al. (2020)
*Serratia* sp. 1–9	Wheat (*Triticum aestivum*)	Greenhouse; isolation and molecular characterization using 16S rRNA; evaluation of drought tolerance and plant growth-promoting traits; evaluation of plant growth dynamics	Drought stress	Auxin; growth in N-free media	Increase of shoot and root dry weight, plant biomass and root	Wang et al. (2014)
*S. plymuthica* MBSA-MJ1	Petunia (*Petunia* × *hybrida*) (), impatiens (*Impatiens walleriana*), and pansy (*Viola* × *wittrockiana*)	*In vitro*; greenhouse; whole-genome sequencing of strain; taxonomy and phylogenetic comparison; evaluation of drought tolerance and plant growth-promoting traits; evaluation of plant growth dynamics	Drought stress	Production of ACC deaminase	Increase of flower number, shoot and root biomass, shoot dry biomass.	Nordstedt and Jones, 2021

To our knowledge, this mini-review is the first review of plant growth promotion by bacteria of the genus *Serratia* spp. The mini-review aims to provide an overview of the protective role of *Serratia* spp. against various abiotic stresses such as salinity, drought, and nutrient deficiency. Based on recent studies, important features of *Serratia* spp. that stimulate plant growth under normal conditions are also discussed. Additionally, the aim of the mini-review is to delineate areas that require further research, particularly more detailed studies elucidating the molecular mechanisms of action of various *Serratia* spp. traits that mitigate abiotic stresses in plants.

## 2 Mini-review methodology

The mini-review analysis was conducted using a variety of keywords such as: abiotic stress, oxidative stress, osmotic stress, ionic stress, salinity stress, drought stress, PGPB, *Serratia*, plant growth-promoting traits, plant physiology, ROS, lipid peroxidation, antioxidant activity, expression of genes, and nutrient imbalance stress. The data were extracted from six databases, namely Google Scholar, Web of Science, Scopus, freefullpdf, Researchgate, and Sciencedirect. Thereby, 98 articles published between 2005 and 2024 were selected. Thence, information was obtained on salinity stress (33 articles), drought stress (24 articles), nutrient uptake (28 articles), and plant growth stimulation under normal conditions (20 articles) by bacteria of genus *Serratia.*

## 3 *Serratia* spp. strains as plant growth-promoting bacteria in normal and nutrient deficiency conditions

To date, a large number of studies has been published on the effects of different *Serratia* sp. strains on plant growth promotion. As previously evidenced, phytohormone production, AHL, ACC deaminase, nitrogen fixation, P and Zn solubilization, and biofilm formation play crucial roles in stimulating plant growth under normal conditions through the activity of *Serratia* spp. strains (Martínez et al., 2018; Hakim et al., 2021; Debnath et al., 2023).

Importantly, studies using bacteria of *Serratia* spp. have been carried out on plants cultivated under different cultivation conditions. For instance, after inoculation of rice plants with *S. glossinae* GS2 and *S. fonticola* GS2, studies conducted in a climate chamber showed a significant increase in plant growth parameters and chlorophyll content (Jung et al., 2017b, 2020). Interestingly, Jung et al. (2017a,b), by liquid chromatography, also showed that the rhizospheric strains *S. glossinae* GS2 and *S. fonticola* GS2 are capable of producing quorum-sensing (QS) signaling molecules (N-hexanoyl-L-homoserine lactone and N-octanoyl-L-homoserine lactone) that play an important role in biofilm formation and plant-microbe interactions. Besides, Zhang et al. (2022), in growth chamber experiment, revealed that *S. marcescens* PLR inoculation increases the expression of auxin biosynthesis genes in *Arabidopsis* roots and the expression of nutrient transporter genes (N, P, K, and S), which in turn promotes lateral root formation and benefits plant growth. In another pot experiment (on wheat), a genomic analysis of *S. marcescens* OK482790 revealed the presence of key nitrogen cycle genes (Hamada and Soliman, 2023). The strain produces lytic enzymes and antimicrobial compounds and reduces ammonia, which suggests its potential role in wheat plant growth. On the other hand, in a greenhouse experiment, Zheng et al. (2022) reported that *S. marcescens* X-45 may promote plant growth by modifying the taxonomic composition of inoculated plants, which probably contributed to the symbiotic nitrogen-fixing effect and consequently to the growth of inoculated plants.

It has also been reported that after the application of *Serratia* spp. strains, not only did the growth parameters of plants such as *Origanum* L., chamomile, and turmeric improve, but also the essential oil content of the plants increased significantly (Alraey et al., 2019; Mostafa et al., 2019; Jagtap et al., 2023). *S. marcescens* AL2-16 also enhanced the growth and biomass of the medical plant *Achyranthes aspera* L. growth (Devi et al., 2016).

Also, there is research where strains of *Serratia* spp. were used to stimulate plant growth under field conditions. For instance, two strains of *S. liquefaciens* (B.AT and B.A10) showed the ability to, e.g., fix nitrogen, produce IAA, siderophores, and EPS, and enhance plant growth and chlorophyll and carotenoid content (Samet et al., 2022). Interestingly, Nascente et al. (2019) conducted a field experiment noting that the efficacy of *Serratia* spp. strains may be dependent on the nitrogen dose. The application of *Serratia* sp. BRM 32114 resulted in a significant increase in rice yield, stomatal conductance in plants, and nitrogen, calcium, and magnesium content in the soil at relatively low nitrogen fertilization rates (0, 20, 40, and 80 kg N ha-1) compared to the control. In contrast, higher amounts of nitrogen (120 kg ha ^−1^) reduced the beneficial effects of BRM 32114 on rice plants compared to the control (Nascente et al., 2019).

In addition, an important PGPB trait is the ability to solubilize insoluble phosphorus compounds in the soil and thus make their bioavailable forms available to plants. Among the mechanisms enabling phosphorus solubilization are the organic acids production (e.g., malic acid, lactic acid, and acetic acid) and the production of phosphatases including acid (ACP, EC 3.1.3.2) and alkaline (ALK, EC 3.1.3.1) (Behera et al., 2017; Barra et al., 2018). To date, many works have described the significant role of PGPB, including strains from the genus *Serratia,* in solubilizing insoluble phosphorus. For example, a statistically significant increase in N, P, and K content in plants was observed after maize inoculation with *Serratia* sp. QW45 (Zhang et al., 2018). Similar results were obtained in a study conducted by Zafar-ul-Hye et al. (2017) where inoculation of wheat seeds with *S. ficaria* W10 (alone or in combination with *Enterobacter cloacae* W6) significantly increased the concentration of N, P, and K in shoots and grains of plants. In addition, the aforementioned phosphate-solubilizing (also EPS- and auxin-producing) bacteria significantly improved growth parameters, spike number, and wheat yield; this fact indicates their potential as a biofertilizer for plants, especially those in nutrient-poor soils (Zafar-ul-Hye et al., 2017). Besides, plant growth stimulation through phosphate solubilization was also observed in strains such as *Serratia* sp. S2, *S. marcescens* CDP-13 (Dogra et al., 2019), *Serratia* sp. KPS-14 (Hanif et al., 2020), *Serratia* sp. LX2 (Guo et al., 2021), and *S. plymuthica* BMA1 (Borgi et al., 2020).

Also, several reports show that *Serratia* sp. S119 can stimulate the growth of peanut and corn due to its ability to solubilize inorganic phosphate, thereby supporting plants in environments low in available P (Anzuay et al., 2017; Ludueña et al., 2018). Ludueña et al. (2023) revealed that the root exudates obtained from plants grown under P-deficient conditions promote colonization and biofilm formation of the strain *Serratia* sp. S119 and stimulate its ability to solubilize phosphate and ACC deaminase activity.

Importantly, bacteria of *Serratia* spp. can also promote growth in nitrogen-poor soils (Zaheer et al., 2016). For instance, inoculation with *Serratia* sp. 5D resulted in a 30.85% increase in a chickpea grain yield in nutrient-poor areas (N, P, K), compared to the uninoculated control (Zaheer et al., 2016). Moreover, in nitrogen-poor cultivation, the strain *Serratia* sp. ZM synthesizes more IAA, thereby supporting root and plant growth (Ouyang et al., 2017).

It has also been documented that *Serratia* spp. strains can be potential candidates for combating Zn deficiency by increasing the bioavailability of the element to plants through various mechanisms (Table 2).

**Table 2:** Effective *Serratia* spp. with Zn solubilizing mechanism.

**Strains of *Serratia* spp.**	**Plants**	**Insoluble Zn form**	**Plant growth promoting traits**	**References**
*S. nematodiphilia* TM 56	Rice (*Oryza sativa* L.)	ZnO, ZnCO_3_, and Zn_3_(PO4)_2_	Zn, P solubilization, IAA production, nitrogen fixation, siderophore production, cellulose degradation, organic acid production	Othman et al. (2022)
*Serratia* sp.	Rice (*Oryza sativa* L.)	ZnSO_4_ and ZnO	Zn solubilization	Idayu (2017)
*Serratia* sp. TM9	Rice (*Oryza sativa* L. cultivar MR219)	ZnSO_4_ and ZnO	Zn solubilization	Idayu et al. (2017)
*S. marcescens*	Rice (*Oryza sativa* L.)	ZnO, ZnCO_3_, and ZnPO_4_	Zn, P solubilization, siderophore production. IAA production; ammonia production, EPS production	Upadhayay et al. (2022)
*S. marcescens* FA-4	Wheat (*Triticum aestivum* L.), Rice (*Triticum aestivum* L.)	ZnO, ZnCO_3_, ZnS	Zn solubilization, ACC-deaminase activity. N2 fixation, EPS activity, siderophore activity, antifungal activity	Abaid-Ullah et al. (2015) and Shakeel et al. (2023)
*S. liquefaciens* FA-2	Wheat (*Triticum aestivum* L.)	ZnO, ZnCO_3_	Zn solubilization, ACC-deaminase activity. N2 fixation, EPS activity, siderophore activity, antifungal activity	Abaid-Ullah et al. (2015)
*Serratia* sp.	Maize (*Zea mays* L.)	ZnO	Zn solubilization, ACC deaminase activity, ammonia production, GA_3_ production	Kour et al. (2019)
*S. marcescens* AF8I1	Carrot (*Daucus carota* L.)	ZnO, [ZnCO_3_]_2_ [Zn(OH)_2_]_3_, ZnCO	Zn, P, K solubilization, IAA, ammonia production, chitinolytic activity, siderophore activity	Fiodor et al. (2023)

In conclusion, *Serratia* spp. demonstrates potential as a plant growth-promoting bacteria (PGPB) with diverse mechanisms. Understanding these mechanisms is crucial for harnessing their full potential in sustainable agriculture. Therefore, further research is needed to determine the mechanisms of, for instance, solubilization of phosphorus, the vast majority of which is unavailable to plants in the soil. Knowledge of whether a *Serratia* spp. strains solubilize phosphorus via the production of organic acids or phosphatases could contribute to more extensive exploitation of the potential of such a strain. Additionally, research on *Serratia* spp. should be conducted under field conditions, as this brings PGPB closer to commercialization.

## 4 Alleviation of salinity stress in plants by *Serratia* spp.

Long-term salinity causes ion toxicity due to increased concentrations of Na^+^ and Cl^−^ ions, which leads to nutrient imbalance and decreased osmotic potential of the soil; such conditions induce oxidative stress (Egamberdieva et al., 2018). More specifically, oxidative stress is induced by the production of toxic ROS mainly including hydrogen peroxide (H_2_O_2_), singlet oxygen (^1^O_2_), superoxide ions superoxide anion (O_2_^•−^), hydroxyl radical (HO^•^), peroxy radical (ROO^•^), alkoxyl radicals (RO^•^), and reactive nitrogen species (RNS) (Gupta et al., 2022; Szechyńska-Hebda et al., 2022). The ROS cause lipid peroxidation, inactivation of antioxidant enzymes (CAT, POD, SOD, APX, GR), reduced photosynthetic rates, membrane permeability and denaturation of DNA, RNA, and proteins, which activates programmed cell death (Nawaz et al., 2020; Mousavi et al., 2022).

Some recent studies have revealed the beneficial effects of *Serratia* spp. strains under saline conditions on various crops such as maize (*Zea mays* L.) (Becze et al., 2021), wheat (*Triticum aestivum* L.) (Desoky et al., 2020), and quinoa (*Chenopodium quinoa* Willd.) (Mahdi et al., 2021). For example, studies under controlled conditions by Becze et al. (2021) showed that after inoculation with *S. fonticola* BB17 at NaCl concentrations of 1–3 g L^−1^, the biomass and chlorophyll a + b content of maize plants increased significantly. Similarly, inoculation of maize with *S. liquefaciens* KM4 with 80 and 160 mM NaCl increased photosynthetic efficiency (chlorophyll a, b, a + b and carotenoids). Interestingly, the authors attributed these results to enhancing the expression of genes mediating the photosynthesis (RuBisCO coding genes: *rbcL* and *rbcS*). Another study also evaluated the effect of strain from *Serratia* spp. on genes expression. El-Esawi et al. (2018) documented that maize inoculated with *S. liquefaciens* KM4 showed significantly less oxidative damage compared to the control group; the strain up-regulated the expression of the most important stress-related genes (CAT, SOD, APX, H^+^-PPase, HKT1, and NHX1), which in turn alleviated the toxicity of Na^+^ and Cl^−^ ions, thus enhancing plant tolerance to salinity. Similar beneficial effects were observed in a study on wheat inoculation with *S. marcescens* M8 at different salinity levels (150 mM and 300 mM NaCl) (Desoky et al., 2020). In this study, increases in enzymatic and non-enzymatic antioxidant activities (CAT, POD, SOD, AsA, GsH, α-TOC) and significant reductions in Na^+^ levels and oxidative stress biomarkers (H_2_O_2_ and O_2_^•−^) alleviated the deleterious effects of salinity stress and enhanced wheat growth.

ACC deaminase is crucial for mitigating salt stress in wheat by *Serratia* spp. strains (Barra et al., 2016; Acuña et al., 2019). Zahir et al. (2009) reported that *S. proteamaculans* M35, exhibiting ACC deaminase activity at different salinity conditions (ES = 1.63–15.0 ds m^−1^), significantly increased biometric parameters of wheat plants and improved K^+^/Na^+^ ratio. The positive effect of ACC deaminase under salinity stress was also showed in a study by Nadeem et al. (2013) where wheat seeds after inoculation with *S. ficaria* W10 were sown in naturally saline fields (EC = 1.0–15.0 dS m^−1^), which significantly increased germination percentage, germination rate, wheat seed rate, and plant yield, compared to the uninoculated control. Additionally, the ACC deaminase producing strain *S. marcescens* CDP-13 minimized oxidative damage, thereby increasing wheat plant tolerance under various levels of salt stress (150 mM, 175 mM and 200 mM NaCl) (Singh and Jha, 2016a). Similar results were obtained using the ACC deaminase-producing strain of *Serratia* sp. SL-12 in wheat grown on saline soil. The authors observed that *Serratia* sp. SL-12 increased plant growth parameters, facilitating nutrient uptake (Singh and Jha, 2016b). Furthermore, the ACC deaminase producing consortium consisting of *S. ficaria* W10, *Pseudomonas putida*, and *P. fluorescens* improved the physiology, growth, and yield characteristics of wheat (Sohaib et al., 2020). Despite the fact that ACC deaminase is known to reduce ethylene levels by degrading ACC and thus reducing the effects of stress on plants, the molecular mechanism of action of *Serratia* spp. strains in alleviating the effects of drought stress in plants is still relatively poorly understood. On the other hand, explanatory studies have emerged showing how halotolerant strain responds to salinity at the level of the proteome. ACC deaminase-producing *S. plymuthica* Sp2 was molecularly studied using a proteomic approach (Novello et al., 2022). Overall, the study revealed that the levels of proteins involved in salt stress responses (4.4% NaCl) were increased, while the levels of proteins involved in metabolism and strand structure were decreased. The main proteins with increased expression in the Sp2 proteome were DsbA, ATP, Porin OmpA, Porin OmpC, and LpoB (Novello et al., 2022).

In addition, it was recently reported that *S. rubidaea* strain ED1, which produces siderophores, cellulase, ammonia, and indole-3-acetic acid (IAA) through its ability to solubilize phosphate and insoluble zinc compounds, increased seed germination and seedling growth of quinoa under salt stress conditions (Mahdi et al., 2021).

Numerous studies have confirmed the potential of *Serratia* spp. strains as biofertilizers promoting plant growth while improving physiological, biochemical, and molecular parameters under salt stress conditions. Nevertheless, we believe that there is a need for further studies of halotolerant *Serratia* spp. strains which have shown tolerance to salinity from 5.8 to 10% NaCl and significant potential in promoting plant growth under normal conditions (George et al., 2013; Hamane et al., 2023; Jagtap et al., 2023). Screening strains of *Serratia* spp. (exhibiting stimulation under normal conditions) for plant growth promotion under saline conditions could increase the list of strains that mitigate the effects of said stress and contribute to the selection of the best ones. Importantly, research is also needed to explain how specific PGP traits affect the expression of genes responsible for mitigating the effects of salinity stress. Such studies should be carried out using omics approach, which will allow a deeper insight into, for example, the plant proteome after the introduction of *Serratia* spp.

## 5 Mitigation of drought stress by *Serratia* spp.

In almost all regions of the world, drought stress is one of the most serious problems recognized as one of the factors limiting crop productivity (Kour and Yadav, 2022). In response to drought stress, plants have lower stomatal conductance, leading to reduced CO_2_ uptake and consequently a decrease in the photosynthesis rate (Saberi Riseh et al., 2021). Plants exposed to drought stress reduce leaf water potential, transpiration rate, and turgor factor, leading to reduced relative water content (RWC) and plant cell damage or death (Kasim et al., 2021).

There are various microorganisms that can effectively help plants under water deficit conditions (Abdelaal et al., 2021). Experiments conducted under drought conditions have shown that *Serratia* spp. strains are capable of effectively stimulating plant growth (Khan and Singh, 2021). In terms of alleviating drought stress, the best studied species of *Serratia* spp. is *S. odorifera.* ACC deaminase-producing strain *S. odorifera* (accession number KC425221) provided tolerance to drought stress by regulating ethylene levels in plants (Ullah et al., 2020; Gul et al., 2023). For instance, a positive effect of *S. odorifera* application was found on wheat in a study by Bangash et al. (2013). Besides, under water deficit conditions, *S. odorifera* (alone or in combination with biocarbon) was able to significantly improve soil nutrition (N, P, and K) and plant growth parameters of barley and maize, compared to the control (Ullah et al., 2020; Gul et al., 2023). The studied strain also significantly increased photosynthetic parameters (chlorophyll a, b, and a + b), NPK content, and SOD, POD, and CAT activities in fresh leaves.

The capacity to mitigate the effects of drought was also observed in other bacteria from *Serratia* spp. The ACC deaminase- and EPS-producing strain *S. marcescens* RRNII not only improved the physiological status and productivity of wheat crops, but also increased micronutrient contents (Zn and Fe) in grains (Khan and Singh, 2021). Interestingly, the EPS produced by the bacteria, under drought stress conditions, provide a microenvironment that retains water, thereby protecting the bacteria from drying out by increasing water availability in the plants (Zhang et al., 2020). The role of EPS-producing bacteria from *Serratia* spp. in promoting growth of maize and rice under drought conditions has also been demonstrated in the studies of Yaseen et al. (2020) and Pang et al. (2020). Furthermore, according to Wang et al. (2012) and Shinde and Borkar (2018), osmolytic proline may also play an important role in the plant response to drought stress by protecting plants from excessive dehydration. Wang et al. (2012) reported that after inoculation of cucumber (*Cucumis sativa* L.) seedlings with a BBS consortium (*Bacillus cereus* AR156, *Bacillus subtilis* SM21, and *Serratia* sp. XY21), leaf proline content increased 4-fold compared to the control. However, in a study by Shinde and Borkar (2018), inoculation of sorghum (*Sorghum bicolor*) seeds with *S. marcescens* L1SC8 and *S. marcescens* L2FmA4 resulted in a significant increase in proline content, which also proved to be beneficial in mitigating the effects of drought stress.

In summary, strains of *Serratia* spp. may help plants tolerate drought through mechanisms such as the production of ACC deaminase, EPS, various phytohormones, increased nutrient uptake, induction of osmolyte, and antioxidant accumulation. However, it is worth adding that the biochemical and molecular mechanisms governing the resistance and tolerance to drought stress induced by *Serratia* spp. strains are still unclear, thus further research is needed. As in the case of mechanisms related to salinity stress, studies using omics methods at the level of the transcriptome or proteome are needed to elucidate the detailed effects of PGP traits on plants under drought conditions.

## 6 Conclusions and future prospective

In sustainable agriculture, *Serratia* spp. holds promise as a valuable tool enhancing both crop yields and quality while aiding plants in managing abiotic stresses. Nevertheless, further research into the mechanisms of action of these bacteria is crucial to fully realize their potential in agricultural practice. As previously mentioned, there is insufficient knowledge regarding the impact of PGP Serratia spp. on the expression of genes associated with growth and protection against abiotic stresses in various plants. We believe that these issues should continue to be investigated, particularly using omics methods. Expanding our knowledge of these mechanisms may also lead to improvements in the effectiveness of *Serratia* spp. strains in important plants, for instance wheat, maize, soya or rice.

Furthermore, studies on the impact of *Serratia* spp. strains on the native microbiota are particularly relevant, especially on the rhizosphere microbiota which belongs to the plant-microbiome axis and seems to be the most significant. To the best of our knowledge, there has been only one publication to date describing this issue, which is insufficient to assess the impact of *Serratia* spp. on the native rhizosphere microbiota. Further studies on a wide range of plants, soils, and different climate types are therefore needed to determine repeatable patterns. Obtaining information on the increase or decrease in abundance of the crucial taxa (e.g., Proteobactera, Actinobacteriota, Acidobacteriota) involved in main soil biochemical processes, may contribute to the development of different optimized formulations with increased efficiency in promoting plant growth. In addition, insights into the native microbiota after application of *Serratia* spp. are also needed to assess bacterial and fungal diversity, the disruption of which may contribute to a decline in soil quality and yield. Research on *Serratia* spp. strains that promote plant growth should also be expanded to determine the potential for biofilm formation, which could optimize rhizosphere colonization.

At the end, it should also be noted that some strains of *Serratia* can be plant and human pathogens, which has been overlooked in the context of research on PGPB. Therefore, prior to field studies, it is vital to examine whether the genomes of PGPB strains contain genes associated with virulence.

## Author contributions

IK: Writing – original draft, Conceptualization. BW: Writing – original draft. JD: Writing – review & editing, Supervision.
